# Supplementary figures and images for: Introgressive hybridization in a Spiny-Tailed Iguana, Ctenosaura pectinata, and its implications for taxonomy and conservation
Source: PeerJ. 2019 Apr 23;7:e6744. doi: 10.7717/peerj.6744 (PMC6485205; doi:10.7717/peerj.6744)

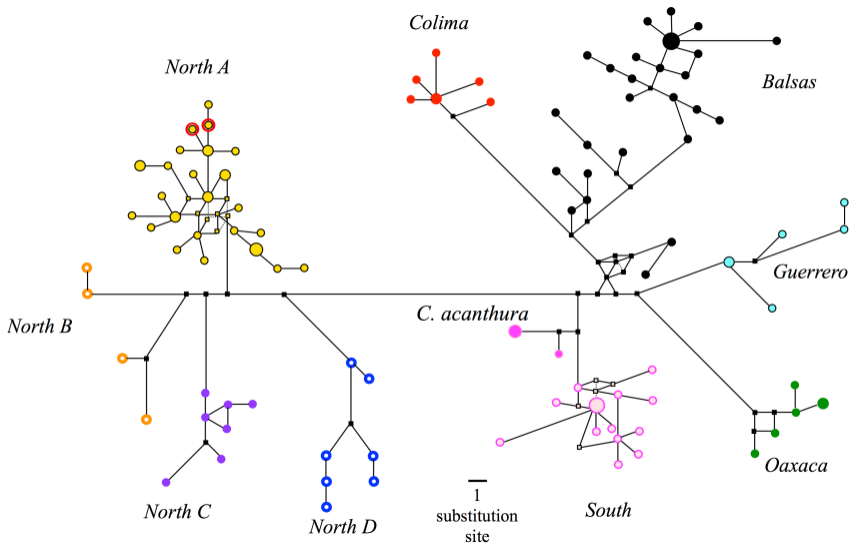

Supplement: Supplemental Information 1 — Calculated with data from Zarza, Reynoso & Emerson (2008, 2011); Haplotypes produced by Zarza Franco (2008) were added to the North A lineage and are highlighted with a red circle. [file peerj-07-6744-s001.pdf]

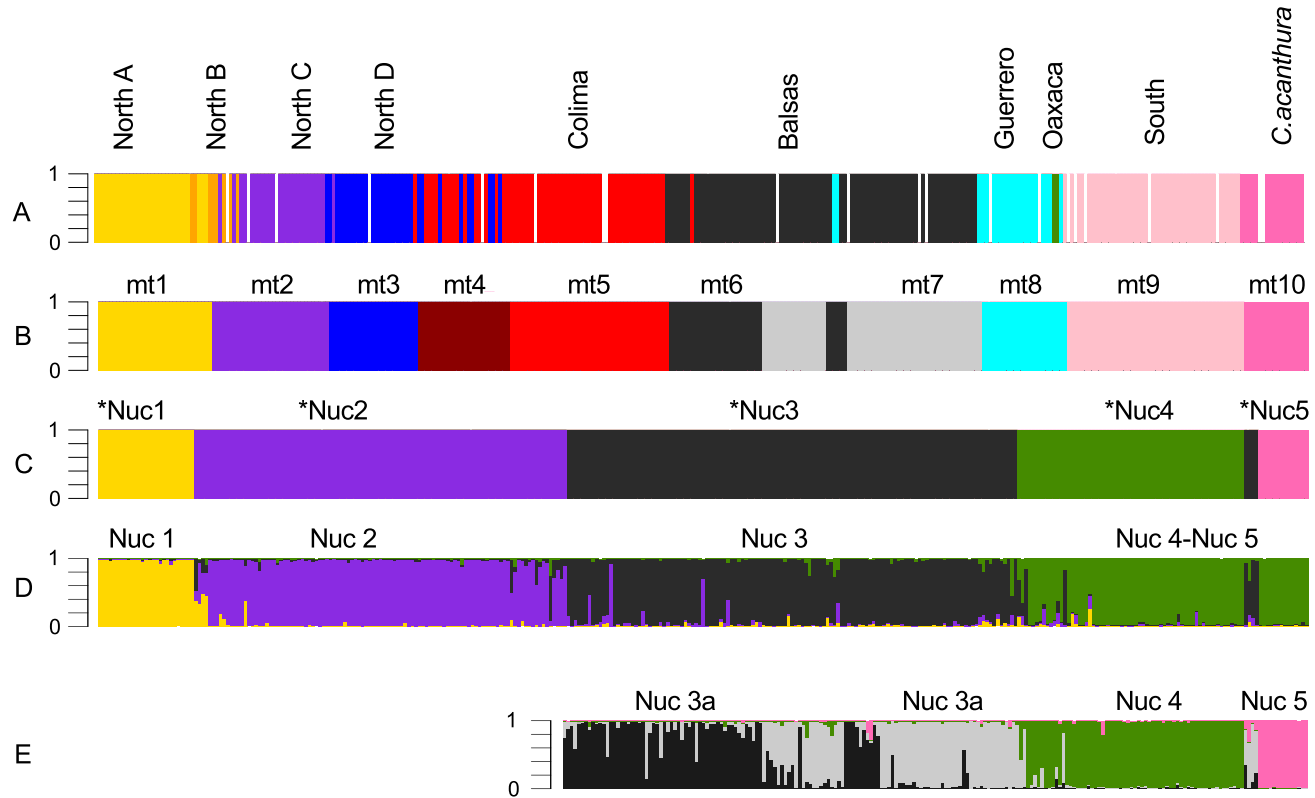

Supplement: Supplemental Information 2 — (A) MtDNA lineage of each individual as defined in haplotype networks calculated by Zarza, Reynoso & Emerson (2008, 2011); (B) SAMOVA mtDNA groups detected under K = 10; (C) microsatellite genotypic cluster defined with SAMOVA under K = 5 and (D) STRUCTURE under K = 4; (E) substructure estimated with STRUCTURE in a reduced data set (South-SS analyses). In STRUCTURE plots, the Y-axis represents proportion of ancestry. As this cannot be calculated with SAMOVA, values are always shown as 1. Each bar represents an individual. White bars are missing data [file peerj-07-6744-s002.pdf]

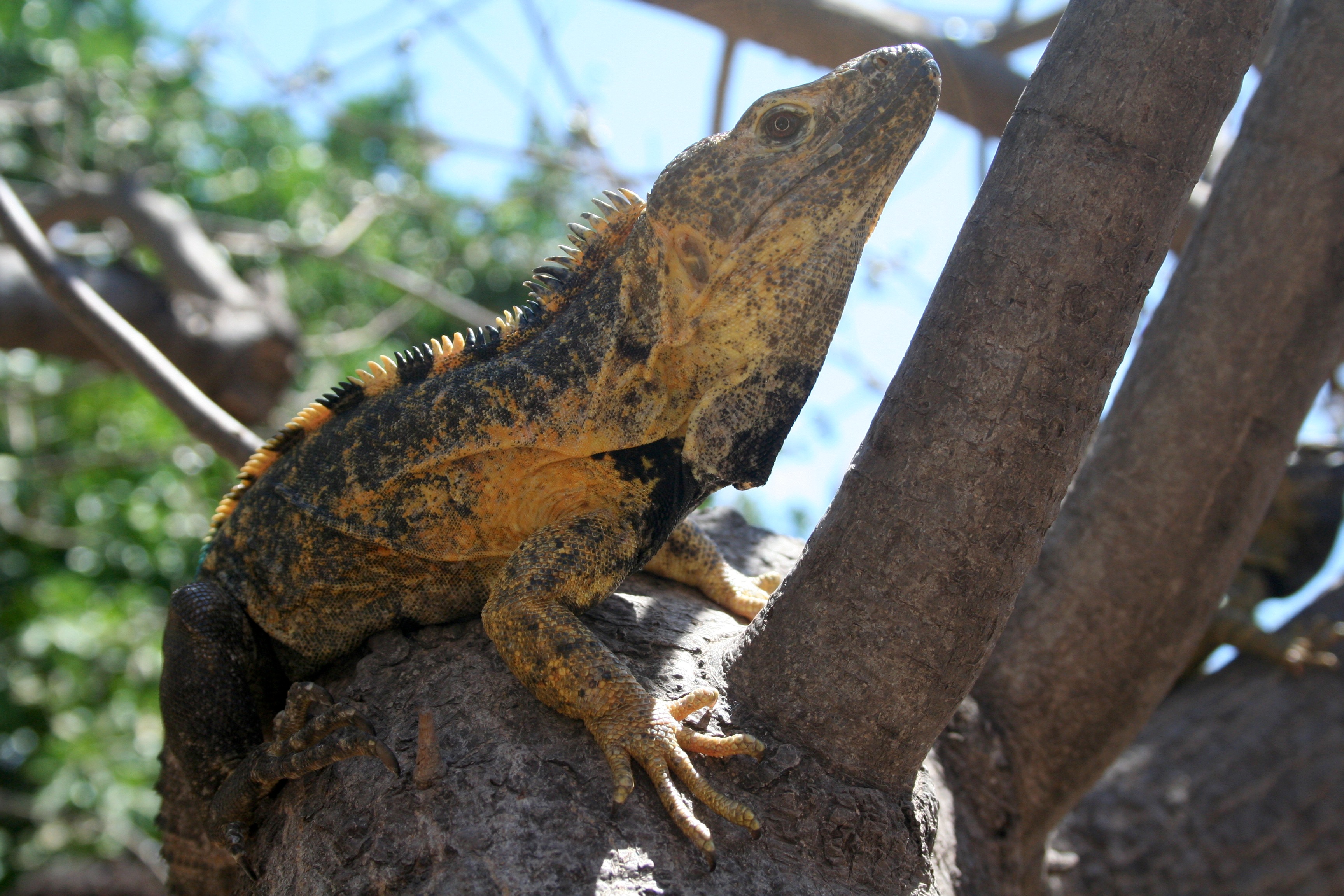

Supplement: Supplemental Information 3 — Here, we propose that populations from northern Mexico are referred as Ctenosaura brachylopha. Photo Credit: Eugenia Zarza. [file peerj-07-6744-s003.jpg]
