# Supplementary material for: Introgressive hybridization in a Spiny-Tailed Iguana, Ctenosaura pectinata, and its implications for taxonomy and conservation
Source: PeerJ. 2019 Apr 23;7:e6744. doi: 10.7717/peerj.6744 (PMC6485205; doi:10.7717/peerj.6744)
Supplement: Supplemental Information 5 [file peerj-07-6744-s005.docx]

**Supplemental information File S2. SAMOVA K associated FCT values, Δ FCT plots; STRUCTURE K likelihoods and Δ K plots.**

Table A. SAMOVA estimated source of variation and fixation indices for mtDNA (above) and microsatellite (below) data under K2-K15. Red font indicates statistically significant values. Bold font highlights the preferred K value suggested by Δ FCT < 0.01.

| **mtDNA** | | | | | | | | | | | | | | |
| --- | --- | --- | --- | --- | --- | --- | --- | --- | --- | --- | --- | --- | --- | --- |
| K | 2 | 3 | 4 | 5 | 6 | 7 | 8 | 9 | **10** | 11 | 12 | 13 | 14 | 15 |
| Variation among groups | 53.24 | 55.75 | 61.21 | 65.29 | 69.64 | 73.06 | 75.72 | 77.64 | 79.28 | 80.22 | 81.23 | 81.90 | 82.54 | 83.18 |
| Variation among populations within groups | 37.12 | 34.38 | 27.33 | 22.91 | 18.16 | 14.68 | 11.95 | 9.94 | 8.12 | 7.17 | 6.00 | 5.27 | 4.51 | 3.85 |
| Variation within populations | 9.64 | 9.87 | 11.46 | 11.80 | 12.20 | 12.26 | 12.33 | 12.42 | 12.59 | 12.62 | 12.77 | 12.83 | 12.95 | 12.97 |
| FCT | 0.53 | 0.56 | 0.61 | 0.65 | 0.70 | 0.73 | 0.76 | 0.78 | 0.79 | 0.80 | 0.81 | 0.82 | 0.83 | 0.83 |
| FSC | 0.79 | 0.78 | 0.70 | 0.66 | 0.60 | 0.54 | 0.49 | 0.44 | 0.39 | 0.36 | 0.32 | 0.29 | 0.26 | 0.23 |
| FST | 0.90 | 0.90 | 0.89 | 0.88 | 0.88 | 0.88 | 0.88 | 0.88 | 0.87 | 0.87 | 0.87 | 0.87 | 0.87 | 0.87 |
| Δ FCT | 0.03 | 0.05 | 0.04 | 0.04 | 0.03 | 0.03 | 0.02 | 0.02 | **0.01** | 0.01 | 0.01 | 0.01 | 0.01 |  |
| **Microsatellites** | | | | | | | | | | | | | | |
| K | 2 | 3 | 4 | **5** | 6 | 7 | 8 | 9 | 10 | 11 | 12 | 13 | 14 | 15 |
| Variation among groups | 29.94 | 16.64 | 19.44 | 21.66 | 22.38 | 23.09 | 23.30 | 23.41 | 23.51 | 23.58 | 23.64 | 23.68 | 23.68 | 23.69 |
| Variation among populations within groups | 15.25 | 12.73 | 8.59 | 6.08 | 5.30 | 4.71 | 4.09 | 3.89 | 3.73 | 3.51 | 3.45 | 3.36 | 3.28 | 3.26 |
| Variation among individuals within populations | 1.08 | 1.39 | 1.42 | 1.42 | 1.42 | 1.42 | 1.43 | 1.43 | 1.43 | 1.43 | 1.43 | 1.43 | 1.44 | 1.44 |
| Variation within individuals | 53.74 | 69.24 | 70.55 | 70.84 | 70.90 | 70.78 | 71.17 | 71.27 | 71.33 | 71.48 | 71.48 | 71.52 | 71.60 | 71.61 |
| FCT | 0.30 | 0.17 | 0.19 | 0.22 | 0.22 | 0.23 | 0.23 | 0.23 | 0.24 | 0.24 | 0.24 | 0.24 | 0.24 | 0.24 |
| FSC | 0.22 | 0.15 | 0.11 | 0.08 | 0.07 | 0.06 | 0.05 | 0.05 | 0.05 | 0.05 | 0.05 | 0.04 | 0.04 | 0.04 |
| FIS | 0.02 | 0.02 | 0.02 | 0.02 | 0.02 | 0.02 | 0.02 | 0.02 | 0.02 | 0.02 | 0.02 | 0.02 | 0.02 | 0.02 |
| FIT | 0.46 | 0.31 | 0.29 | 0.29 | 0.29 | 0.29 | 0.29 | 0.29 | 0.29 | 0.29 | 0.29 | 0.28 | 0.28 | 0.28 |
| Δ FCT | -0.13 | 0.03 | 0.02 | **0.01** | 0.01 | 0.00 | 0.00 | 0.00 | 0.00 | 0.00 | 0.00 | 0.00 | 0.00 |  |

Figure A Fixation indices under K=2 – K=15 obtained with SAMOVA for mtDNA data (left) and Δ FCT (FCT K +1 - FCT K) (right). K was selected as above. Red dot indicates the preferred K value.


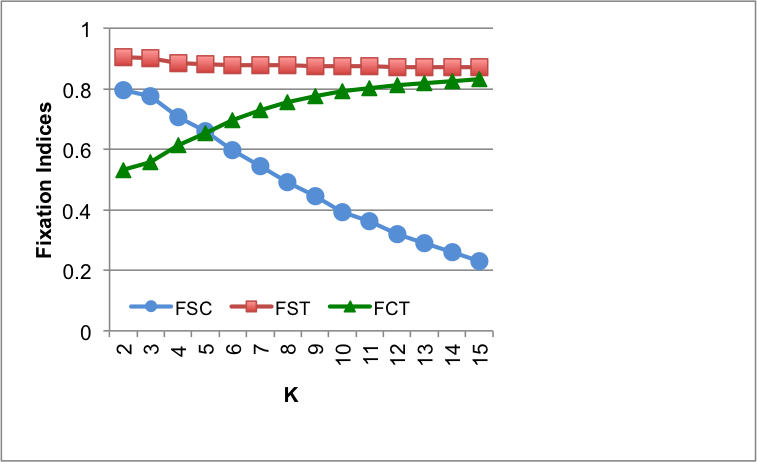

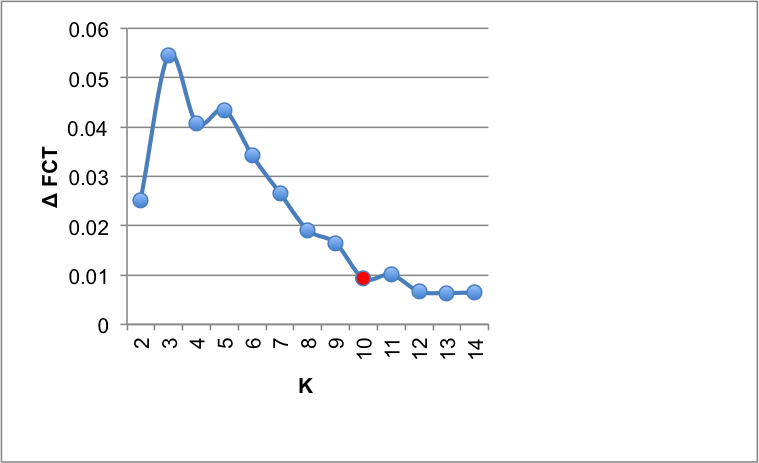


Figure B Fixation indices under K=2 – K=15 obtained with SAMOVA for microsatellite data (left) and Δ FCT (FCT K +1 - FCT K) (right). K was selected if a <1% change was observed in FCT values between K and K+1, as indicative of FCT starting to plateau. Red dot indicates the preferred K value.


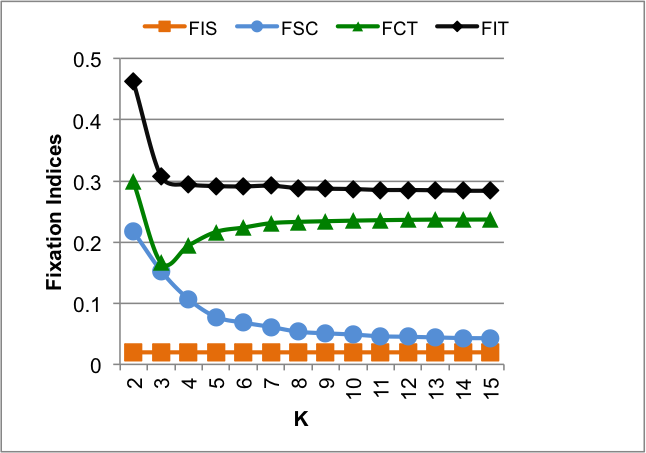

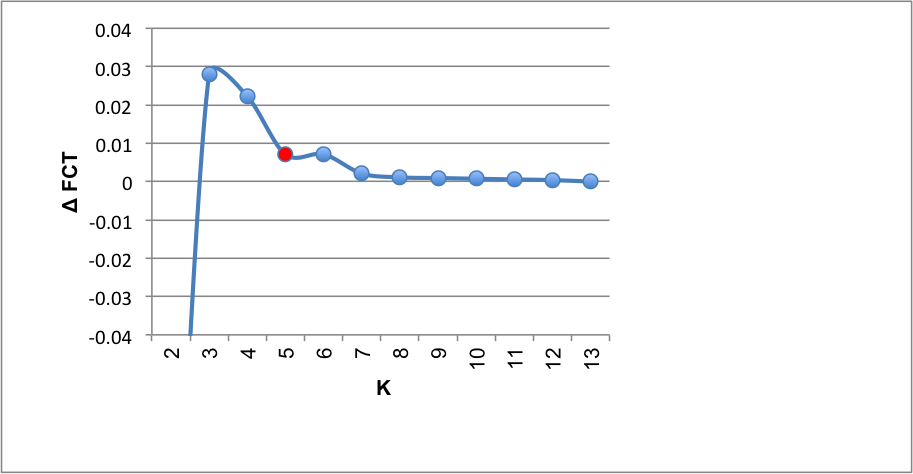


Figure C. Plot of mean likelihood and standard deviation (SD) values for K=2 – K=10 (left) and Δ K ([1]; right) obtained with STRUCTURE. Notice the wide SD for K=6 which probably led to Δ K peaking at K=7. Graphs produced with Structure Harvester Web v0.6.94 [2].

Figure D. Plot of mean likelihood and SD values for K =2 – K= 10 (left) and Δ K (right) after removing two outlier runs, obtained with STRUCTURE. Notice the now narrower SD for K=6. Δ K peaks at K=4. Graphs produced as above.

Figure E. Plot of mean likelihood and SD values for K =2 – K= 6 (left) and Δ K (right) of a subsample of localities (17-49) to investigate substructure. Δ K peaks at K=4 Graphs produced as above

References

1. Evanno G, Regnaut S, Goudet J. Detecting the number of clusters of individuals using the software STRUCTURE: a simulation study. Mol Ecol. 2005;14: 2611–2620.

2. Earl DA, vonHoldt BM. STRUCTURE HARVESTER: a website and program for visualizing STRUCTURE output and implementing the Evanno method. Conserv Genet Resour. 2012;4: 359–361. doi:10.1007/s12686-011-9548-7
