# Supplementary material for: Introgressive hybridization in a Spiny-Tailed Iguana, Ctenosaura pectinata, and its implications for taxonomy and conservation
Source: PeerJ. 2019 Apr 23;7:e6744. doi: 10.7717/peerj.6744 (PMC6485205; doi:10.7717/peerj.6744)
Supplement: Supplemental Information 6 [file peerj-07-6744-s006.pdf]

Supplemental information Table S1. Localities pairwise FST values obtained with Arlequin 3.5.

Distance method: pairwise differences. Red font indicates statistically significant values. Significance level=0.05.

Gray intensity refelcts genetic differenciation intenisty, with darker tones denoting greater genetic differenctiation

	1	2	3	4	5	6	7	8	9	10	11	12	13	14	15	16	17	18	19	20	21	22	23	24
1																								
2	0.02																							
3	0.03	0.01																						
4	0.17	0.25	0.19																					
5	0.20	0.28	0.21	0.13																				
6	0.18	0.24	0.19	0.13	0.11																			
7	0.20	0.25	0.20	0.14	0.10	0.00																		
8	0.20	0.24	0.18	0.15	0.09	-0.03	-0.05																	
9	0.22	0.28	0.21	0.16	0.08	-0.04	-0.02	-0.04																
10	0.18	0.22	0.18	0.17	0.13	0.00	0.01	-0.03	-0.01															
11	0.23	0.28	0.23	0.19	0.11	0.00	-0.01	0.00	-0.07	0.02														
12	0.22	0.26	0.22	0.17	0.12	0.02	0.02	-0.01	-0.04	0.03	0.01													
13	0.22	0.27	0.22	0.14	0.10	0.00	-0.01	-0.02	-0.03	0.04	0.00	0.01												
14	0.23	0.27	0.23	0.20	0.11	0.04	0.01	-0.01	-0.09	0.03	0.00	0.01	0.01											
15	0.24	0.26	0.22	0.16	0.14	0.02	0.01	-0.01	-0.04	0.02	0.01	0.03	-0.01	0.02										
16	0.25	0.26	0.22	0.16	0.15	0.07	0.03	0.01	0.01	0.06	0.03	0.03	0.03	0.05	0.01									
17	0.29	0.29	0.28	0.26	0.29	0.19	0.15	0.15	0.17	0.17	0.15	0.15	0.14	0.14	0.12	0.08								
18	0.30	0.30	0.28	0.23	0.28	0.16	0.13	0.11	0.15	0.16	0.15	0.14	0.12	0.13	0.09	0.08	0.04							
19	0.34	0.34	0.31	0.28	0.32	0.20	0.18	0.17	0.21	0.21	0.18	0.20	0.16	0.20	0.14	0.09	0.04	0.01						
20	0.33	0.31	0.29	0.28	0.32	0.19	0.17	0.14	0.20	0.20	0.18	0.18	0.14	0.18	0.12	0.09	0.04	-0.01	0.00					
21	0.33	0.33	0.30	0.28	0.29	0.20	0.17	0.15	0.21	0.21	0.19	0.19	0.14	0.17	0.15	0.11	0.05	0.04	0.04	0.02				
22	0.32	0.32	0.29	0.26	0.29	0.20	0.16	0.15	0.23	0.21	0.19	0.19	0.15	0.18	0.14	0.11	0.04	0.02	0.04	0.01	-0.01			
23	0.37	0.35	0.32	0.30	0.40	0.23	0.21	0.20	0.23	0.23	0.23	0.21	0.19	0.19	0.15	0.14	0.11	0.01	0.09	0.06	0.07	0.02		
24	0.37	0.34	0.33	0.35	0.40	0.27	0.23	0.23	0.29	0.27	0.25	0.24	0.21	0.24	0.18	0.12	0.06	0.05	0.03	0.00	0.02	0.00	-0.02	
25	0.34	0.32	0.31	0.30	0.34	0.24	0.21	0.22	0.23	0.25	0.20	0.21	0.18	0.20	0.17	0.13	0.05	0.06	0.06	0.04	0.08	0.06	0.09	0.01
26	0.41	0.44	0.40	0.37	0.41	0.26	0.23	0.26	0.30	0.28	0.24	0.25	0.21	0.26	0.20	0.14	0.15	0.11	0.08	0.09	0.15	0.11	0.14	0.03
27	0.31	0.35	0.30	0.29	0.32	0.18	0.15	0.19	0.18	0.21	0.15	0.17	0.13	0.17	0.14	0.11	0.11	0.08	0.08	0.08	0.09	0.09	0.16	0.11
28	0.38	0.38	0.35	0.34	0.39	0.24	0.21	0.24	0.27	0.26	0.22	0.23	0.17	0.22	0.17	0.14	0.11	0.08	0.07	0.05	0.10	0.09	0.15	0.03
29	0.29	0.27	0.26	0.23	0.29	0.20	0.17	0.15	0.18	0.20	0.18	0.17	0.16	0.17	0.15	0.12	0.03	0.06	0.09	0.06	0.09	0.09	0.10	0.07
30	0.30	0.29	0.28	0.25	0.30	0.19	0.19	0.18	0.20	0.20	0.17	0.18	0.16	0.18	0.13	0.08	0.04	0.07	0.05	0.04	0.09	0.05	0.06	0.01
31	0.33	0.32	0.30	0.29	0.32	0.21	0.20	0.18	0.23	0.23	0.20	0.20	0.16	0.20	0.16	0.12	0.08	0.08	0.06	0.04	0.06	0.05	0.11	0.03
32	0.31	0.32	0.29	0.28	0.25	0.10	0.04	0.07	0.06	0.10	0.04	0.01	0.02	-0.03	0.03	0.01	-0.08	-0.04	0.05	0.04	-0.05	-0.04	-0.06	0.05
33	0.36	0.36	0.33	0.33	0.34	0.20	0.17	0.16	0.21	0.20	0.18	0.14	0.13	0.14	0.14	0.09	0.03	0.02	0.07	0.03	-0.01	0.03	0.08	0.05
34	0.32	0.34	0.30	0.28	0.29	0.18	0.15	0.17	0.21	0.21	0.18	0.13	0.15	0.16	0.17	0.16	0.17	0.13	0.20	0.15	0.15	0.11	0.13	0.16
35	0.24	0.26	0.23	0.22	0.26	0.15	0.14	0.15	0.16	0.18	0.15	0.18	0.13	0.16	0.14	0.12	0.12	0.09	0.10	0.08	0.09	0.09	0.14	0.10
36	0.23	0.21	0.20	0.27	0.28	0.19	0.18	0.17	0.21	0.19	0.21	0.19	0.18	0.17	0.19	0.18	0.16	0.15	0.21	0.17	0.17	0.17	0.19	0.19
37	0.39	0.38	0.36	0.41	0.42	0.30	0.29	0.30	0.34	0.28	0.30	0.26	0.26	0.25	0.27	0.27	0.21	0.21	0.27	0.24	0.22	0.22	0.25	0.26
38	0.34	0.37	0.32	0.31	0.28	0.16	0.18	0.15	0.18	0.19	0.18	0.17	0.16	0.18	0.17	0.17	0.23	0.21	0.25	0.23	0.23	0.24	0.27	0.28
39	0.35	0.34	0.31	0.31	0.33	0.19	0.19	0.16	0.21	0.20	0.21	0.16	0.14	0.16	0.16	0.15	0.11	0.10	0.14	0.11	0.10	0.13	0.18	0.16
40	0.31	0.30	0.30	0.33	0.37	0.22	0.24	0.22	0.23	0.21	0.24	0.20	0.23	0.22	0.22	0.21	0.21	0.21	0.25	0.22	0.25	0.25	0.26	0.26
41	0.33	0.32	0.31	0.36	0.37	0.23	0.24	0.22	0.22	0.22	0.23	0.16	0.21	0.20	0.22	0.20	0.18	0.20	0.25	0.22	0.23	0.25	0.28	0.27
42	0.38	0.38	0.36	0.35	0.36	0.26	0.26	0.24	0.27	0.27	0.27	0.24	0.24	0.26	0.24	0.22	0.22	0.22	0.23	0.23	0.25	0.27	0.32	0.28
43	0.39	0.38	0.33	0.35	0.36	0.26	0.26	0.25	0.23	0.26	0.26	0.23	0.23	0.24	0.22	0.17	0.19	0.22	0.21	0.23	0.24	0.27	0.36	0.30
44	0.49	0.47	0.46	0.56	0.57	0.40	0.42	0.44	0.52	0.37	0.45	0.43	0.43	0.46	0.40	0.43	0.41	0.39	0.44	0.41	0.46	0.46	0.54	0.52
45	0.36	0.37	0.35	0.34	0.32	0.24	0.22	0.21	0.23	0.23	0.21	0.21	0.20	0.21	0.19	0.17	0.19	0.21	0.22	0.22	0.24	0.26	0.33	0.28
46	0.39	0.38	0.36	0.37	0.41	0.27	0.29	0.28	0.32	0.27	0.30	0.27	0.26	0.29	0.29	0.27	0.26	0.26	0.28	0.27	0.27	0.29	0.34	0.34
47	0.38	0.38	0.34	0.35	0.39	0.22	0.20	0.21	0.25	0.24	0.21	0.20	0.17	0.22	0.15	0.14	0.12	0.09	0.11	0.04	0.12	0.10	0.15	0.05
48	0.58	0.58	0.55	0.64	0.64	0.50	0.51	0.54	0.64	0.47	0.54	0.51	0.50	0.54	0.48	0.48	0.47	0.43	0.45	0.45	0.50	0.49	0.60	0.52
49	0.46	0.44	0.42	0.54	0.57	0.36	0.38	0.40	0.45	0.33	0.40	0.38	0.39	0.41	0.35	0.36	0.31	0.34	0.36	0.32	0.41	0.42	0.51	0.45

	25	26	27	28	29	30	31	32	33	34	35	36	37	38	39	40	41	42	43	44	45	46	47	48
26	0.09																							
27	0.04	0.07																						
28	0.01	-0.01	0.02																					
29	0.04	0.18	0.12	0.12																				
30	0.02	0.07	0.07	0.04	0.08																			
31	0.06	0.08	0.07	0.05	0.10	0.03																		
32	0.01	0.20	-0.01	0.07	0.01	-0.01	0.01																	
33	0.05	0.17	0.08	0.08	0.06	0.05	0.04	-0.22																
34	0.16	0.16	0.11	0.14	0.17	0.13	0.14	-0.18	0.02															
35	0.07	0.10	0.02	0.02	0.13	0.07	0.09	0.01	0.09	0.11														
36	0.19	0.32	0.19	0.22	0.16	0.16	0.20	0.03	0.15	0.18	0.11													
37	0.21	0.38	0.22	0.25	0.22	0.21	0.24	0.00	0.16	0.19	0.18	0.06												
38	0.26	0.35	0.23	0.27	0.24	0.22	0.24	0.09	0.21	0.21	0.19	0.12	0.13											
39	0.11	0.27	0.11	0.16	0.11	0.12	0.14	0.01	0.04	0.15	0.12	0.04	0.06	0.12										
40	0.23	0.32	0.21	0.27	0.23	0.21	0.25	0.18	0.23	0.28	0.20	0.11	0.11	0.18	0.10									
41	0.22	0.35	0.21	0.27	0.20	0.21	0.24	0.06	0.16	0.22	0.20	0.09	0.03	0.14	0.05	0.00								
42	0.23	0.34	0.26	0.28	0.23	0.24	0.26	0.22	0.24	0.31	0.24	0.21	0.22	0.16	0.09	0.14	0.12							
43	0.21	0.38	0.26	0.27	0.21	0.21	0.24	0.25	0.26	0.35	0.22	0.23	0.24	0.21	0.11	0.18	0.14	0.06						
44	0.46	0.60	0.53	0.57	0.38	0.45	0.47	0.58	0.53	0.55	0.43	0.45	0.51	0.50	0.47	0.36	0.43	0.36	0.50					
45	0.22	0.34	0.24	0.28	0.22	0.21	0.25	0.21	0.24	0.33	0.22	0.18	0.21	0.16	0.09	0.12	0.11	0.04	0.06	0.43				
46	0.26	0.37	0.27	0.31	0.27	0.27	0.27	0.24	0.28	0.33	0.24	0.21	0.18	0.24	0.15	0.09	0.10	0.18	0.22	0.45	0.18			
47	0.08	0.04	0.08	0.06	0.13	0.09	0.09	0.08	0.08	0.13	0.11	0.26	0.30	0.31	0.19	0.26	0.26	0.29	0.30	0.45	0.29	0.35		
48	0.47	0.63	0.60	0.60	0.44	0.48	0.48	0.70	0.62	0.64	0.49	0.62	0.66	0.63	0.61	0.51	0.61	0.46	0.61	0.50	0.53	0.59	0.46	
49	0.38	0.61	0.46	0.52	0.27	0.38	0.42	0.67	0.51	0.55	0.35	0.37	0.49	0.47	0.41	0.28	0.34	0.28	0.42	0.17	0.35	0.41	0.39	0.62
